# Giving Oncolytic Viruses a Free Ride: Carrier Cells for Oncolytic Virotherapy

**DOI:** 10.3390/pharmaceutics13122192

**Published:** 2021-12-18

**Authors:** Alberto Reale, Arianna Calistri, Jennifer Altomonte

**Affiliations:** 1 Department of Molecular Medicine, University of Padua, 35121 Padua, Italy; alberto.reale@studenti.unipd.it (A.R.); arianna.calistri@unipd.it (A.C.); 2 Department of Internal Medicine II, Klinikum Rechts der Isar, Technical University of Munich, 81675 Munich, Germany

**Keywords:** oncolytic virus, virotherapy, carrier cells, cancer immunotherapy

## Abstract

Oncolytic viruses (OVs) are an emerging class of therapeutics which combine multiple mechanisms of action, including direct cancer cell-killing, immunotherapy and gene therapy. A growing number of clinical trials have indicated that OVs have an excellent safety profile and provide some degree of efficacy, but to date only a single OV drug, HSV-1 talimogene laherparepvec (T-Vec), has achieved marketing approval in the US and Europe. An important issue to consider in order to accelerate the clinical advancement of OV agents is the development of an effective delivery system. Currently, the most commonly employed OV delivery route is intratumoral; however, to target metastatic diseases and tumors that cannot be directly accessed, it is of great interest to develop effective approaches for the systemic delivery of OVs, such as the use of carrier cells. In general, the ideal carrier cell should have a tropism towards the tumor microenvironment (TME), and it must be susceptible to OV infection but remain viable long enough to allow migration and finally release of the OV within the tumor bed. Mesenchymal stem cells (MSCs) have been heavily investigated as carrier cells due to their inherent tumor tropism, in spite of some disadvantages in biodistribution. This review focuses on the other promising candidate carrier cells under development and discusses their interaction with specific OVs and future research lines.

## 1. Introduction

The last century has indeed witnessed substantial progress in the so-called “war on cancer” [1]. Much of this is owed to primary prevention, including lifestyle modifications (e.g., a reduction in the number of smokers) and vaccines against the oncogenic human papillomavirus (HPV), and to early detection, associated with screening programs that are very cost-effective for some tumors (breast, cervix, colon adenocarcinoma) in the appropriate age groups [2,3,4]. The improvement of surgical techniques also allows resection of more advanced primary tumors and isolated metastases [5]. When early detection by screening or curative surgery are not possible, the medical treatment of cancer has also improved, but the picture in this field is significantly more blurred [1]. Very effective pharmacological treatments are available for some hematologic malignancies, such as Hodgkin’s lymphoma and acute lymphoid leukemia, and targeted therapies have changed the natural course of chronic myeloid leukemia and significantly altered the prognosis of some subsets of solid tumors such as EGFR-positive lung carcinoma. However, the overall prognosis of common epithelial cancers when diagnosed at a disseminated stage remains poor. In fact, the mortality associated with sporadic but aggressive tumors that are difficult to diagnose early, such as glioblastoma and pancreatic ductal adenocarcinoma, has remained stable over the years, thus requiring innovative therapeutic approaches [6].

In light of recent cancer immunotherapy breakthroughs, especially the immune checkpoint inhibitors (ICIs), it has also become clear that the response to immunotherapy varies widely. Melanoma has “traditionally” been the tumor that is most vulnerable to immunotherapeutic strategies, since the employment of high-dose recombinant interleukin 2 (IL-2) in the 1980s, and it is also quite sensitive to ICIs [7]. In particular, following a combination of inhibitors of two checkpoint molecules, programmed cell death 1 (PD1) and CTLA-4, an objective response rate of over 50% was seen in melanoma, although at the cost of significant side effects, most notably autoimmune diseases [8]. Apart from melanoma, responses have also been observed in non-small cell lung carcinoma (NSCLC) and renal carcinoma [9,10].

It is interesting to note that many tumors do not show any clinical response to ICIs despite the presence of antigens that can be potentially recognized by the immune system [11]. While this is partially due to the down-regulation of antigen-presenting molecules (MHC-I) on the surface of tumor cells and other mechanisms of antigen masking, these tumors are also surrounded by a tumor microenvironment (TME) that is markedly immunosuppressive and excludes lymphocytes while including a mainly myeloid immune infiltrate [12]. The role of the TME is exemplified by cases of melanoma that are poorly susceptible to ICIs and are characterized by a reduced number of lymphocytes (a feature that is also called, somewhat emphatically, an “immunologic desert”).

Oncolytic viruses (OVs) are attenuated viruses that exploit defects in cellular antiviral pathways that are often present in cancer cells. OVs are usually based on human pathogens, including adenoviruses, herpes simplex virus type 1 (HSV-1), vaccinia virus (VACV) and measles virus (MV), although several animal viruses, such as vesicular stomatitis virus (VSV) and Newcastle disease virus (NDV), are also under development as OV vectors. In recent years, these viruses have been recognized as a class of immunotherapeutics, due to their ability to elicit an immune response against the tumor [13]. In particular, some preclinical and clinical data indicate that OVs can turn immunologically “cold” tumors into “hot” ones by enhancing the infiltration of lymphocytes and thereby improving the efficacy of ICIs. In clinical trials, OVs have demonstrated an excellent safety profile, though effectiveness has been somewhat disappointing [14]. To date, the only OV which has been approved for clinical use is talimogene laherparepvec or T-Vec, a virus based on HSV-1 with a deletion of the neurovirulence gene Δγ34.5 and of the Us12 gene, which reduces antigen presentation in infected cells and is armed with a human granulocyte-monocyte colony stimulating factor (hGM-CSF) [15]. Importantly for the aim of this review, T-Vec is delivered by multiple intratumoral injections once every two weeks. Different OVs have been armed with therapeutic genes that enhance cancer cell death (proapoptotic genes) or promote an antitumoral immune response (immunotherapeutic genes). Although the length of foreign sequences that can be inserted in the viral genome is dependent on the viral vector and is largest in HSV-1 and vaccinia virus (VACV) [16,17], many OVs support therapeutic genes, including adenoviruses, vesicular stomatitis virus, Maraba virus, measles virus, orthoreoviruses and influenza viruses [18,19,20,21,22,23]. Most investigated OVs, their mechanisms of action and the main clinical trials involving OVs, along with possible strategies to improve their efficacy, have been reviewed elsewhere [13].

Despite advancements in OV development over the years [24], the most appropriate delivery mode for OVs is still a matter of debate. In theory, the intravenous injection of antitumoral drugs seems to be ideal in order to target the primary tumor, as well as systemic metastases and micrometastases which are below the limit of detection of current diagnostic techniques. However, apart from safety issues, there are many drawbacks to the systemic injection of OVs, the most important being the effect of the immune system, which threatens to remove or neutralize attenuated OVs before they reach the tumor [25]. This effect was observed with different viruses including HSV-1 [26] and adenoviruses [27] in preclinical models, in which most of the injected virus was sequestered in the liver and spleen [28]. While this pattern of accumulation might not limit the intravenous treatment of hepatic tumors [29], it negatively affects the treatment of other deep-seated tumors. The neutralization of virions in the bloodstream is particularly relevant in the case of viruses with a high seroprevalence in the population, such as HSV-1 [30]. Therefore, intratumoral injection has become the method of choice for OV delivery, especially since the immunologic mechanism of action was widely accepted. The problem of targeting metastases was addressed by relying on the “in situ vaccine” effect [31]. According to this hypothesis, the lytic effect of the virus is limited to the primary tumor in which it is injected, but the immune response against tumor-associated antigens (TAAs) will also be effective against the uninjected masses. Indeed, such a response was observed in clinical trials of T-Vec against melanoma but only in a limited number of cases (9% response in visceral metastases in the OPTiM trial) [32], which led to the investigation of synergism with ICIs. In this setting, the possibility of using carrier cells has emerged as a promising method to achieve the systemic delivery of OVs [33].

**Figure 1:**
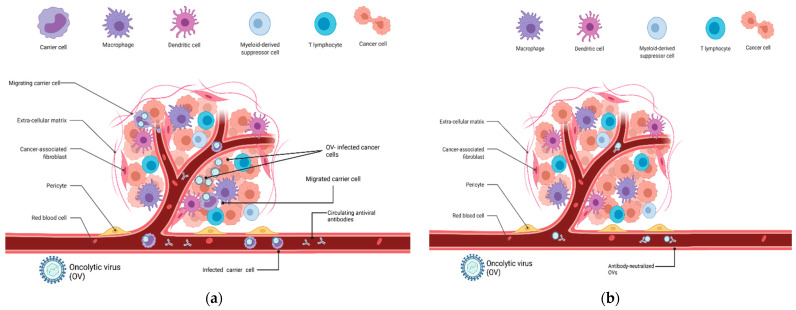
Bioavailability of an oncolytic virus to the tumor microenvironment following intravenous injection in the presence of pre-existing antibodies: (**a**) OV-infected carrier cells migrate into the TME, where they release viral particles, even in the presence of antiviral antibodies in the bloodstream; (**b**) cell-free virions in the bloodstream are neutralized by antibodies and cannot infect cancer cells.

Carrier cells can be loaded with the OVs ex vivo and then injected intravenously, and it has been demonstrated in animal models that they can effectively shield OVs from antibody-mediated neutralization and nonspecific uptake (Figure 1). This would greatly improve biodistribution and potentially enhance safety, since lower systemic doses would be needed in order to achieve a sufficient amount of virus delivered to the tumor. Nevertheless, developments in this approach are in a relatively early phase, and the direct intratumoral delivery of OVs is still standard practice, both in basic and clinical research. This review will focus on the different cell types that have been proposed as OV carriers and the many unresolved issues in the intricate interplay between carrier cells, the host immune system and the tumor microenvironment. Until now, the cell type that was by far the most investigated as carriers for OVs was mesenchymal stem cells (MSCs) [34,35]. However, since numerous reviews describing the use of MSCs for the delivery of OV therapies are already available [33], this review will focus on other types of OV carrier cells, mostly comprising immune cell subsets, which have been less frequently reviewed.

Despite the potential of MSCs, there are many good reasons to also consider other carrier cell candidates. As MSCs have immunosuppressive properties, they may actually be counterproductive to the anti-tumor immune response, which the OV therapy aims to achieve. MSCs were also shown to have pharmacokinetic challenges, accumulating mainly in the lungs of experimental animals following intravenous injection, probably due to their dimensions. These problems resulted in some research groups trying to use MSCs for intratumoral delivery, which can make sense in some particular instances but mostly seems to contradict the rationale for the use of carrier cells.

## 2. Carrier Cells for Oncolytic Virus Delivery

While the use of carrier cells is an attractive approach to potentially improve the pharmacokinetics and biodistribution of OVs, the ideal cell type to employ for this function is a matter for debate. The optimal carrier cells for the delivery of oncolytic viruses should possess three essential features: (1) they must have a tropism towards tumors; (2) they must be able to either internalize the virus or allow virions to stably attach to their cell membrane; (3) they must maintain viability for a sufficient time to allow distribution in the bloodstream and delivery of the viral cargo to the tumor site(s). Furthermore, although somewhat an issue of debate, there is a strong rationale for the use of autologous cells in order to avoid any issues of rejection. Beyond this, a lot of uncertainty arises, and there is no consensus in the field. What is the ideal kind of carrier cell to achieve the efficient systemic delivery of OVs? Traditionally, it has been assumed that an antiviral immune response should be considered as a negative factor because it induces viral clearance before all cancer cells within the mass have been killed. This is also a factor in the choice of mesenchymal stem cells (MSCs), which have immunosuppressive properties, in many studies on carrier cells and OVs [33] (see paragraph below). Although the inhibition of antiviral immune responses could be beneficial for enhancing the therapeutic effect of the OV, a general immune suppression could dampen the virus-mediated immune-stimulatory effects against the cancer, which would be counterproductive and severely hamper an important mechanism of the therapy. Therefore, the use of immune-suppressive MSCs as part of an oncolytic virus regimen should be carefully considered and tested. It is likely desirable to achieve a fine tuning that allows the virus to replicate in the tumor without dampening the downstream adaptive antitumoral immune response [36].

As already mentioned, most preclinical studies, and the few clinical trials which employed carrier cells as a means of OV delivery so far (Table 1), have employed MSCs [33]. Nevertheless, other cells, including neural stem cells (NSCs) [37], monocytes [38] and T lymphocytes [39], have also been investigated as viral carriers and show potential in this function. These non-MSC cell types and their potential as carrier cells for OV delivery will be the focus of the following sections.

### 2.1. T Lymphocytes

T lymphocytes represent attractive candidates as carrier cells for OV delivery due to their ability to circulate freely in the bloodstream and home to their tumor targets, as well as their potential to provide synergistic therapeutic effects via their cytotoxic effector functions. Even naïve activated T cells have been shown to successfully shield OVs from neutralization and nonspecific uptake while delivering them to the tumor bed. In the case of oncolytic measles virus (MV) therapy, it was shown that a virus loaded onto activated T cells could be transferred to tumor cells, even in the presence of neutralizing anti-MV serum in vitro and in vivo, indicating successful shielding conferred by the approach [39]. Interestingly, the expression of the fusogenic envelope proteins of MV on the surface of infected T cells allowed for the heterofusion of the lymphocytes with the tumor cells, which facilitated the transfer of infectious virions and subsequent lysis of the tumor cells. Similarly, oncolytic reovirus was shown to be protected from pre-existing antiviral immunity when loaded onto T cells, which effectively delivered the virus to B16 melanoma tumors in vivo and mediated an anti-tumor immune response and long-term protection against the tumor [40].

Although Newcastle disease virus (NDV) may be unable to replicate within T lymphocytes, it can bind to the T cell surface via its hemagglutinin-neuraminidase attachment protein, which recognizes cell surface sialic acid-containing receptors that are present on glycoproteins or glycolipids, followed by membrane fusion via the viral F protein. In this way, it was speculated that NDV can “hitchhike” on intravenously applied T cells for delivery and transfer to tumor cells. Proof-of-concept was demonstrated in vitro, whereby oncolytic NDV was attached to the surface of activated peripheral blood-derived T cells and shown to be transferred to human MCF-7 breast carcinoma cells, leading to subsequent oncolysis [41].

In a further demonstration of the utility of antigen-nonspecific T cells as OV carriers, Qiao and colleagues investigated the use of naïve T cells to chaperone oncolytic vesicular stomatitis virus (VSV) to lymphoid organs and, thereby, eradicate metastases [42]. In this interesting approach, it was shown that VSV loaded onto naïve T cells could effectively eliminate primary B16 melanoma lesions, as well as lymph node and spleen metastases, even in virus-immune mice [42]. The group later went on to show that the efficacy of the adoptive transfer of antigen-specific OT-I T cells could be enhanced by loading them with oncolytic VSV in the same B16 tumor model [43]. Here, it was demonstrated that tumor-specific T-cell activation and tumor trafficking was even enhanced by the pre-infection of the T cells with VSV prior to delivery, leading to an effective combinatorial approach.

Antigen-specific T lymphocytes have been utilized for the shielding of a variety of oncolytic virus vectors, including VSV, herpes simplex virus type 1 (HSV-1), adenovirus and vaccinia virus (VV) [44,45,46,47]. Tumor-infiltrating lymphocytes (TILs) are a particularly interesting subset, as they have a natural propensity to infiltrate the tumor bed and do not require additional transduction with a T cell receptor or CAR for tumor targeting. Furthermore, the use of autologous TILs provides a personalized approach, which can potentially target a variety of specific tumor-associated antigens expressed within the patient’s own tumor. Santos and colleagues have recently reported on the systemic delivery of an armed adenovirus vector (TILT-123) using TILs isolated from human ovarian cancer and hamster pancreatic cancer as carriers [47]. They hypothesized that loading an optimized OV vector onto TILs would allow for the delivery of the viral therapeutic to the tumor site, and that the strategy would provide a means for the administration of both therapeutic components in one self-amplifying product, which was then demonstrated in various in vivo models [47].

Yotnda et al. utilized a novel approach, in which tumor-specific cytotoxic T lymphocytes (CTLs) were modified to express the adenoviral E1 gene under the control of the activation-dependent CD40 ligand in order to induce infectious adenovirus production specifically when the CTLs were exposed to HLA-matched tumor antigen-expressing target tumor cells [48]. This not only represents an effective combination therapy and OV delivery approach, but it also provides a clever mechanism for restricted viral dissemination specifically at the target tumor site. The benefit of antigen-specific lymphocytes over naïve T cells was further demonstrated using the oncolytic strain F HSV1 mutant, R3616, which carries a deletion of both copies of the virulence factor γ34.5, which is essential for viral replication in neurons [49], loaded onto lymphocytes harvested from mice that had developed an antitumor immunity. R3616 adsorbed onto antigen-specific lymphocytes mediated in significantly improved responses in mice bearing peritoneally-disseminated tumors, compared to R3616 adsorbed onto naïve lymphocytes or with either monotherapy [50].

In addition to the isolation of autologous antigen-specific T lymphocytes, T cells can also be engineered ex vivo to express a T cell receptor (TCR) or chimeric antigen receptor (CAR) in order to confer tumor-specific cytotoxic effector functions. The recent marketing approval of CAR T cells makes this a particularly attractive approach for OV delivery. In fact, it was recently demonstrated that loading HER2-specific CAR T cells with low doses of VSV or oncolytic vaccinia virus (vvDD) does not interfere with receptor expression or function, and either the RNA or DNA virus could be transferred to target tumor cells using either mouse or human T cells [46]. As an alternative to the CAR T cell approach, we have recently reported on the use of TCR-transduced CD8^+^ central memory T cells as a delivery vehicle for oncolytic VSV, resulting in an effective combination therapy in a xenograft model of acute myeloid leukemia (AML) in mice. Interestingly, in addition to the viral delivery and potent cytotoxic effector functions provided by the central memory subset of TCR T cells, it was shown that the approach substantially improved the safety of intravenously applied VSV compared to the use of a naked virus [45].

Despite the growing body of evidence in support of the use of lymphocytes as OV carriers, one important factor to consider is that various immunosuppressive mechanisms within the tumor microenvironment can mean exclusion of T lymphocytes from the tumor [12]. By design, the function of OV carrier cells is dependent on the ability of those cells to extravasate from the tumor vasculature and infiltrate into the tumor mass. Therefore, the utility of T cells as OV carriers may be limited to those tumors which are not characterized as immune deserts. Additionally, various approaches that are under development to improve lymphocyte trafficking into tumors [51,52] could be applied in OV-loaded T cell regimes in order to pre-sensitize the tumor to the adoptive T cell transfer and concomitantly improve OV delivery. Furthermore, the potential cytotoxic effects of the OV on the lymphocyte would need to be characterized for each viral vector to be utilized in order to avoid the lymphocytes being lysed and releasing their viral cargo before reaching the tumor.

In summary, T lymphocytes, whether naïve, TILs or engineered CAR or TCR T cells, represent promising cell carriers of oncolytic viruses that can shield against antiviral neutralizing antibodies and deliver to the tumor bed, potentially also providing cytotoxic effector functions to synergize with the viral oncolysis. Additional evidence that virus replication within the tumor further enhances T cell-mediated therapeutic effects provides an added rationale for the combination approach. We are likely to see increasing numbers of examples of these approaches in the next few years, as adoptive T cell therapies and oncolytic viruses make their way into routine clinical practice and as new approaches to enhance lymphocyte extravasation and infiltration into tumors are developed.

### 2.2. Myeloid Cells

An assortment of myeloid cells infiltrate the TME of most solid tumors [53]. These include tumor-associated macrophages (TAMs) [54], tumor-associated neutrophils (TANs) [55], dendritic cells and immature myeloid-derived suppressor cells (MDSCs) [56]. Myeloid cells are actually a functional and important part of the TME, where they are actively recruited by chemokines and cytokines and where they play a role in secreting tumorigenic growth factors and dampening the adaptive immune response.

This explains why infiltration by myeloid cells is a conserved feature in many solid tumors, even those with scarce or no lymphocytic infiltrate [12]. For example, the presence of TAMs is so fundamental that new therapeutic strategies have been designed to target these cells to increase the sensitivity of tumors to both immunotherapy and chemotherapy [55]. As a consequence, TAMs or their circulating precursors (monocytes) are attractive carrier cells for OVs because it is unlikely that cancer cells can develop a resistance to treatment by excluding TAMs from the TME. Furthermore, autologous monocytes can be easily recovered in large numbers from peripheral blood and are amenable to differentiation in vitro into macrophages or dendritic cells. Therefore, it seems that some myeloid cells (with the possible exception of neutrophils, which are difficult to maintain ex vivo for OV infection) have almost ideal characteristics as carrier cells. Nevertheless, there remain few reports of monocytes or other myeloid cells being utilized as OV carriers.

Buñuales et al. investigated primary human monocytes and a Syrian hamster monocyte/macrophage cell line (HM-1) [57] as carrier cells for an oncolytic adenovirus (oAdV) in nude mice with human tumors (HuH hepatocarcinoma cell line) and immunocompetent hamsters with hamster pancreatic tumors, respectively [38]. Freshly isolated human monocytes improved the biodistribution of oAdV in nude mice by intravenous injection, while the injection of HM-1 hamster cells resulted in the accumulation of oAdV in the liver. Biodistribution in vivo was measured with a luciferase-expressing adenoviral vector. The authors concluded that the selected carrier cells were not suitable for systemic delivery in an immunocompetent animal model and chose to use HM-1 cells as carriers for an intratumoral delivery of oAdV. In this setting, they showed that intratumoral HM-1-mediated OV administration allowed for repeated therapeutic gene expression after multiple injections, which was more efficient than after injections of naked virus. However, a limitation of this study is that reportedly, even uninfected macrophages from the HM-1 cell line failed to infiltrate the pancreatic tumor, thus not mimicking the real biological behavior of TAMs. Utilizing primary monocytes instead of a macrophage cell line could have potentially led to an improved infiltration.

Peng et al. [58] used dendritic cells to deliver measles virus to an immunocompromised murine model of human myeloma. Immature dendritic cells (iDCs) were selected due to their efficiency in the transmission of oncolytic measles viruses to myeloma cells. Briefly, primary human monocytes were collected and differentiated into iDCs in vitro, infected with an oncolytic measles virus expressing luciferase and injected in the tail vein of SCID mice harboring subcutaneous KAS 6/1 tumors. The authors showed that luciferase activity could be detected in tumors 48 h after the injection. By loading carrier cells with measles viruses expressing a red fluorescent protein (RFP), it was possible to demonstrate that infection was transmitted to tumor cells in vivo. Furthermore, in a systemic myeloma model (SCID mice intravenously injected with KAS 6/1 cells) iDCs loaded with measles virus extended survival but were not curative. This study provided proof-of-concept that monocyte-derived cells can specifically deliver an OV to a tumor, though it did not use an immunocompetent animal model.

A second approach employing DCs as carriers for OV therapy was reported by Ilett et al. [40,59]. Here, DCs were shown to internalize oncolytic reovirus and shield it from inactivation by neutralizing antibodies. Furthermore, it was demonstrated that reovirus-loaded DCs retained their functionality with regards to the phagocytic and T-cell priming potential.

Eisenstein et al. [60] employed MDSCs as delivery vectors for oncolytic vesicular stomatitis virus (VSV) in Balb/c and C57BL/6 mice with implantation of MCA26 colon cancer and Lewis lung carcinoma (LCC) cells, respectively. Different subsets of MDSCs exist in both mice and humans and they are enriched in the blood of patients and experimental animals with cancer. MDSCs are essentially immature myeloid cells with an immunosuppressive phenotype, thus they can be classified according to their lineage of origin as monocytic and granulocytic [56]. In this study, monocytic Ly6C+ MDSCs were isolated from the bone marrow of mice with MCA26 and LCC tumors and loaded with different recombinant VSVs (rVSVs), including a virus with a reporter gene (EGFP), an oncolytic VSV with an altered matrix protein and one expressing the murine gammaherpesvirus M3 protein to delay viral clearance.

The study was quite complex and evaluated different loading conditions (simple MDSC infection versus loading assisted by non-neutralizing anti-VSV antibodies) and different tumor models, including intrahepatic MCA26 cell implantation. It could be demonstrated that VSV-loaded MDSCs improved biodistribution to tumors, protected mice from VSV-induced neurotoxicity, reduced tumor growth and improved survival. Finally, with some conditions, long-term survivors were also achieved.

Although the concept is slightly different, the attempt to use antibody-neutralized oncolytic T3D reovirus, which is presumably internalized by monocytes in vivo and then delivered to tumors, is also noteworthy [61]. The direct loading of monocytes with reovirus was limited, to the best of our knowledge, to in vitro models [61,62]. Finally, it should be mentioned that many myeloid carrier cells are immunosuppressive, which could potentially be counterproductive to the immunotherapeutic effect of oncolytic virotherapy. However, in the aforementioned study by Eisenstein et al., it was demonstrated that VSV infection modulated the activation profile of MDSCs towards an immunogenic phenotype [60]. Furthermore, autologous circulating myeloid cells will naturally accumulate in the tumor bed, making them ideal candidates to deliver OVs to tumor sites.

### 2.3. Neural Stem Cells

While this review focuses mainly on cells of the immune system, neural stem cells are also interesting carrier cells for the treatment of intracranial tumors. In particular, an immortalized human neural stem cell (NSC) line was used for the intracranial delivery of a replication-competent oAdV in athymic mice with intracranial tumors formed by primary human glioblastoma multiforme cells [63]. While this approach does not exploit carrier cells for systemic administration, it takes advantage of their migratory capabilities to “chase” glioma tumor cells that can diffusely infiltrate the brain parenchyma. A clinical trial for the delivery of an oAdV to newly diagnosed glioma patients is also currently ongoing (NCT03072134).

## 3. Discussion

In our opinion, a major hurdle to the clinical translation and market entry of oncolytic virotherapy is the lack of simple, clinician- and patient-friendly administration methods that are effective in the tumor-specific delivery of viral vectors. While a lot of effort has been (correctly) focused on the generation of elaborate, genetically engineered OVs with enhanced selectivity and/or therapeutic gene expression, it is difficult to imagine that, in real-world clinical practice, large numbers of cancer patients can be treated by intratumoral injection of therapeutic viruses. It is not by chance that the only approved OV, talimogene laherparepvec, is used against tumors of the skin (melanoma), which can be easily injected every two weeks, as per the standard talimogene therapeutic schedule [64]. In the case of deep-seated tumors, which unfortunately include most of the deadliest human cancers, intratumoral injection requires interventional, imaging-guided or surgical procedures that are difficult, expensive and often painful to apply in routine clinical practice, especially if multiple injections are needed (the most striking case is probably that of intracranial tumors). Furthermore, as already discussed in the introduction, if the neoplasm is metastatic, it becomes almost impossible to inject all masses, and one needs to rely solely on the immunologic effect of the OV.

Therefore, the rationale behind the use of carrier cells for the targeted, systemic delivery of OVs is indeed convincing and intriguing, and it might have the potential to overcome the main barriers to the virotherapeutic treatment of tumors with a poor prognosis. While some research groups recognized the potential of immune cells, chiefly T lymphocytes and secondarily myeloid cells, proof-of-concept studies were not followed by therapeutic refinement and a progression towards clinical trials. We propose that, in order to bring forward a real integration between carrier cells and oncolytic virotherapy, the researchers’ attention should be focused on autologous cells that can be easily recovered from the patient without lengthy ex vivo culture or differentiation steps. Furthermore, genetic engineering of OVs could be further exploited to enhance the “collaboration” between viruses and carrier cells, for example to boost carrier cell migration into the tumor bed or to achieve prolonged release of OVs from carriers without cytopathic effects. Even if carrier cells have a very high tropism for tumors, it is very likely that they will also accumulate in non-tumoral tissue, which limits the viral dose delivered to the tumor and puts the healthy tissue at risk of viral infection and replication. Therefore, if OV-loaded carrier cells have a tropism towards a specific organ (for example, the lung or the liver) it may be necessary to modify the virus in order to make the normal cells from that organ specifically resistant to infection.

In conclusion, as OV-based therapeutic approaches make their way into routine clinical practice, the need for novel strategies to improve the efficacy of intravenous applications becomes more and more relevant. By combining OV therapies with immune cell-based carrier systems, the opportunity to improve the bioavailability of OVs and enhance tumor-specific uptake can be combined with adoptive cell transfer to synergize the direct oncolytic effect with effector functions of the carrier cell. We believe that these approaches are highly promising and that we will be seeing more examples of immune cell carriers for OV therapies in clinical studies in the near future.

Although oncolytic virotherapy has been explored in different forms for almost a century, the last 20 years have marked a huge progress due to refined genetic engineering techniques, better understanding of the mechanism of action (especially its immunologic side) and the accumulation of data from clinical trials [13,64]. Now it is time for this therapy to “come of age” and become a real therapeutic option for more tumors with a dire prognosis. Carrier cells can be an essential piece of the puzzle.

## Figures and Tables

**Table 1:** Clinical trials involving mesenchymal stem cells (MSCs) as carriers for oncolytic viruses (as of December 2021). Abbreviations: AdV Adenovirus. CELYVIR: bone marrow-derived autologous MSCs infected with ICOVIR5 (oncolytic AdV). AloCELYVIR: bone marrow-derived allogeneic MSCs infected with ICOVIR5 (oncolytic AdV).

Trial	Target Disease	Oncolytic Virus	Results
EudraCT Number: 2008-000364-16	Pediatric solid tumors	ICOVIR5 (AdV) CELYVIR	Trial ended prematurely
NCT 02068794	Ovarian cancer	Measles virus	Recruiting
NCT 01844661	Miscellaneous metastatic tumors	ICOVIR5 CELYVIR	Completed in 2016—results not available
EudraCT Number: 2019-001154-26	Extracranial solid tumors	ICOVIR5 AloCELYVIR	Ongoing
NCT03896568	Recurrent high-grade glioma	AdV, DNX-2401	Recruiting
NCT05047276 (phase I/II)	Metastatic Uveal Melanoma	ICOVIR5 AloCELYVIR	Trial not yet recruiting

## Data Availability

Not applicable.
